# The Rarity in the Rarity: Presentation of Three Cases of Cutaneous Carcinosarcoma with Clinical and Histopathological Insights

**DOI:** 10.3390/dermatopathology11030022

**Published:** 2024-07-15

**Authors:** Gerardo Cazzato, Anna Colagrande, Valentina Caputo, Giuseppe Ingravallo, Eliano Cascardi, Francesco Fortarezza, Emanuela Bonoldi, Franco Rongioletti

**Affiliations:** 1 Section of Molecular Pathology, Department of Precision and Regenerative Medicine and Ionian Area (DiMePRe-J), University of Bari “Aldo Moro”, 70124 Bari, Italy; anna.colagrande@gmail.com (A.C.); giuseppe.ingravallo@uniba.it (G.I.); eliano.cascardi@policlinico.ba.it (E.C.); 2 U.O. Anatomia Patologica, ASST Grande Ospedale Metropolitano Niguarda, 20162 Milan, Italy; valentina.caputo@ospedaleniguarda.it (V.C.); emanuela.bonoldi@ospedaleniguarda.it (E.B.); 3 Surgical Pathology and Cytopathology Unit, University Hospital of Padova, 35100 Padova, Italy; francesco.fortarezza@aopd.veneto.it; 4 Dermatology Clinic, Vita-Salute San Raffaele University, 20132 Milan, Italy; rongioletti.franco@hsr.it

**Keywords:** cCS, heterologous, MCC, pilomatrix carcinoma, BCC, porocarcinoma, rare skin tumors

## Abstract

A cutaneous carcinosarcoma (cCS) is a rare and aggressive skin cancer characterized by both carcinomatous (epithelial) and sarcomatous (mesenchymal) components, making it a biphasic tumor. Despite its occurrence in various organs, a cCS is exceptionally rare in the skin, predominantly affecting older males. The etiology of a cCS is unclear, but it may originate from a single progenitor cell capable of dual differentiation or from a collision of carcinoma and sarcoma cells. Clinically, a cCS presents as a rapidly growing, painful, ulcerated nodule or plaque on sun-exposed skin, with a high risk of local invasion and metastasis. Histopathologically, a cCS includes various epithelial components, such as squamous cell carcinoma and basal cell carcinoma, along with undifferentiated sarcomatous components resembling atypical fibroxanthoma. The tumor may also exhibit heterologous differentiation like angiosarcomatous or rhabdomyosarcomatous features. We present three cases of a cCS, highlighting their clinical and histological characteristics and comparing them with previously reported cases. Understanding a cCS is complicated by its rarity and diverse presentation, emphasizing the need for further research to elucidate its pathogenesis and optimal management.

## 1. Introduction

A cutaneous carcinosarcoma (cCS) (or metaplastic carcinoma of the skin or carcinoma with heterologous differentiation) is a rare and aggressive type of skin cancer that presents both carcinomatous (epithelial) and sarcomatous (mesenchymal) components [1]. This dual nature classifies it as a biphasic tumor, meaning it has two distinct types of malignant cells coexisting within the same biopsy [1,2]. Although a CS can be present in organs such as the respiratory tract, gastrointestinal tract, breast, and uterus, a cCS is an exceptionally rare condition, with few cases documented in the medical literature, and typically affects older adults, with a higher prevalence in males than females [3]. The rarity of this tumor makes it challenging to establish definitive demographic profiles or risk factors beyond those commonly associated with skin cancers, such as prolonged UV exposure [4]. The precise mechanism of cCS development remains unclear, with the prevailing hypothesis suggesting that it originates from a single progenitor cell capable of differentiating into both epithelial and mesenchymal lineages [5]. Alternatively, some researchers propose that the tumor may arise from a collision between an existing carcinoma and a sarcoma, although this is less commonly accepted [5]. Patients with a cCS typically present with a rapidly growing, ulcerated nodule or plaque on sun-exposed areas of the skin, such as the head, neck, and extremities [6], and the lesion is often painful and may bleed or become infected. Due to its aggressive nature, the tumor can invade surrounding tissues and metastasize to distant sites if not treated promptly [6,7]. Histopathologically, a cCS can be composed of different epithelial and mesenchymal components, of which the first one can be represented by a squamous cell carcinoma (SCC), basal cell carcinoma (BCC), Merkel cell carcinoma (MCC), pilomatrix carcinoma, spiradenocarcinoma, porocarcinoma, and also a trichoblastic carcinoma [6]. Regarding the sarcomatous component, usually, the cells are undifferentiated, similar to these one constituting Atypical Fibroxanthoma (AFX), with epithelioid and spindle morphology, brisk and atypical mitotic activity, and with foci of necrosis [7]. Furthermore, the heterologous component can include angiosarcomatous, leiomyosarcomatous, rhabdomyosarcomatous, chondroblastic, or osteoblastic differentiation [8,9].

In this paper, we present three cases of different kinds of cCSs that occurred during our pathological practice, summarize the most important clinical and histological features, and discuss the previous similar cases already published in the literature.

## 2. Case Presentation

### 2.1. Case 1

**Figure 1:**
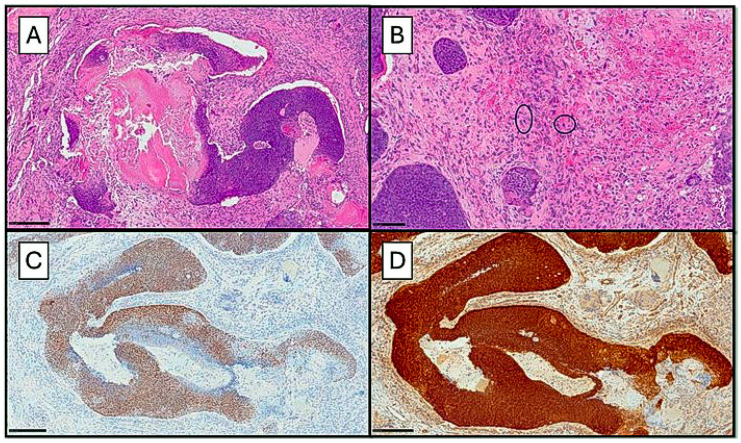
(**A**) Histological photomicrograph showing a neoplasm constituted by an epithelial basaloid component and some shadow cells, with irregular architecture intermingled with mesenchymal elements (H&E, original magnification 10×); (**B**) Photomicrograph showing the sarcomatous component characterized by atypical spindle cells with numerous mitotic figures (black circles) (H&E, original magnification 20×); (**C**) Immunohistochemical preparation for an anti-p40 antibody: note the diffuse nuclear positivity in the basaloid component (immunohistochemistry for anti-p40 antibody, original magnification 10×); (**D**) Immunohistochemical preparation for an anti-β-Catenin antibody: note the diffuse cytoplasmic and nuclear positivity in both basaloid and sarcomatous component (immunohistochemistry for anti-β Catenin antibody, original magnification 10×).

A 64-year-old attended the General Surgery Unit of the Polyclinic of Bari for the appearance of a neoformation of the right elbow, of which the patient was unable to report the timing of onset, but which was clinically hard to the touch, compact, and not painful. It was decided to perform an excisional biopsy, and the sample was sent to Pathological Anatomy. After sampling, processing, cutting, and preparing the slides, the lesion was analyzed under an optical microscope (OM). From a histopathological point of view, the lesion was composed of two different components. The epithelial component was characterized by solid aggregates and cords of immature basaloid cells (with basophilic cytoplasm, nucleoli, and dark chromatin), which infiltrated the dermis and subcutaneous tissue and presented a certain number of shadow cells (Figure 1A). Also, there was a high number of mitotic figures, sometimes atypical, and these cells expressed nuclear and cytoplasmatic staining for β-Catenin (Figure 1D). The sarcomatous component was characterized by spindle atypical cells with mitosis and was closely intermingled with the epithelial component (Figure 1B). Furthermore, p40 was positive in the basaloid cells (Figure 1C), and Vimentin was positive in the mesenchymal component Finally, smooth muscle actin (SMA) was partially positive in the sarcomatous component, and p53 was positive in both epithelial and sarcomatous cells. A diagnosis of a pilomatrical CS was made. Following that, the patient had the surgical exeresis area enlarged to ensure there was no remaining tumor. Computed Tomography (CT) did not show any other localization.

Figure 1A–D summarizes the histological and immunohistochemical features of the case 1.

### 2.2. Case 2

**Figure 2:**
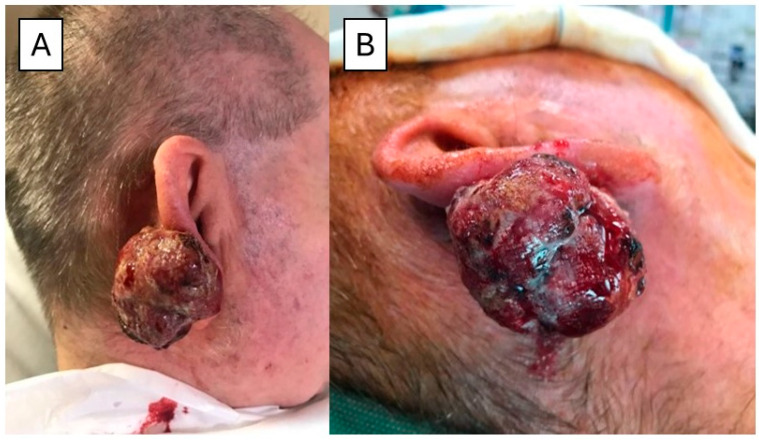
(**A**,**B**) Clinical view of the 5 cm bulky and rapidly growing mass of the right helix with hemorrhage and necrosis.

**Figure 3:**
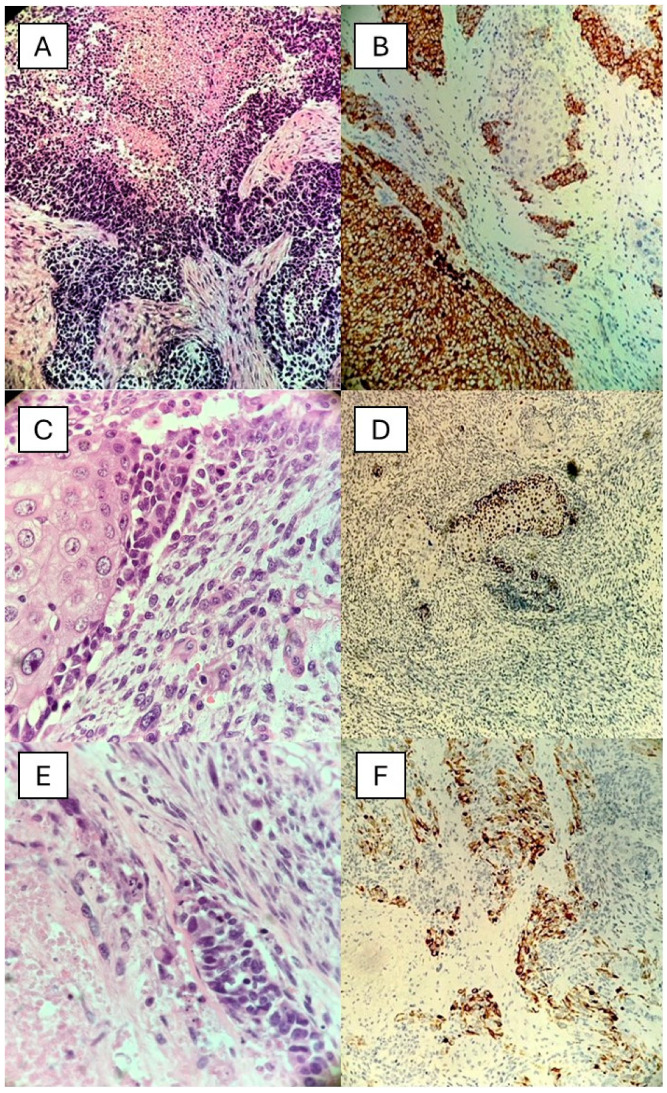
(**A**) Histological photomicrograph showing the MCC component characterized by the typical features with some foci of necrosis (H&E, original magnification 10×); (**B**) Immunohistochemical preparation for an anti-CK20 antibody: note the positivity in dot-like localization of the tumor’s MCC cells (immunohistochemistry for anti-CK20, original magnification 10×); (**C**) Higher magnification of a field showing the SCC component in the neoplasm (H&E, original magnification 20×); (**D**) Immunohistochemical preparation for an anti-p40 antibody: note the nuclear positivity of the SCC component (immunohistochemistry for anti-p40, original magnification 20×); (**E**) Higher magnification of a field showing rhabdomyoblastic differentiation in the neoplasm (H&E, original magnification 20×); (**F**) Immunohistochemical preparation for an anti-Desmin antibody: note the cytoplasmic positivity of rhabdoid component (immunohistochemistry for anti-Desmin antibody, original magnification 10×).

A 75-year-old man with a history of chronic sun exposure presented with a 5 cm bulky and rapidly growing mass of the right helix (Figure 2A,B). The patient had a diagnosis of lung adenocarcinoma pT1b pN2 in 2015, treated with Vinorelbine + Cisplatin, and in 2016 had a progression of disease in the lung and brain treated from 01/2018 with Nivolumab. From a histological point of view, the lesion was composed of different components: Merkel cell carcinoma (MCC) (30%) (Figure 3A), squamous cell carcinoma (SCC) (20%) (Figure 3C), homologous sarcomatoid component (40%), and rhabdomyosarcomatous component (10%), respectively (Figure 3E). Immunohistochemically, the MCC component was positive for CK20 dot-like (Figure 3B), Chromogranin A (CgA), and Synaptophysin (Syn), while the SCC component was positive for p40 (Figure 3D). The homologous sarcomatoid component was positive for Vimentin, and the rhabdomyosarcomatous component was positive for Desmin (Figure 3F) and Myogenin.

Taking everything into account, a diagnosis of Merkel Cell Carcinosarcoma was made. In November 2017, an Ultrasound Scan (US) scan was performed, and a parotid and laterocervical lymphadenopathy were detected, making a lymphadenectomy necessary. Histopathological examination revealed lymph node metastases (7/12 intraparotideal lymph nodes, 2/13 laterocervical lymph nodes), and for this reason, an adjuvant RT was administered.

### 2.3. Case 3

**Figure 4:**
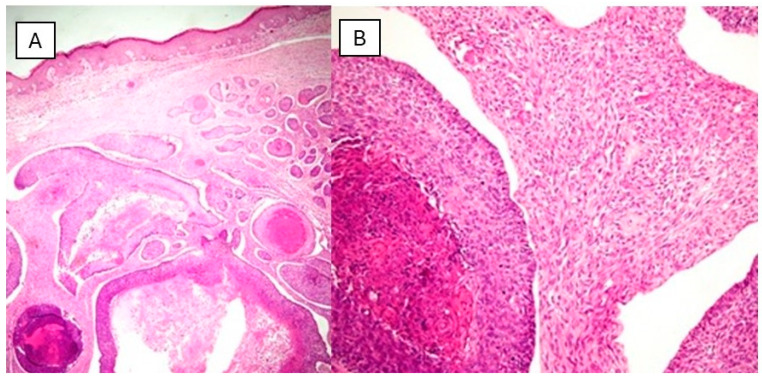
(**A**) Histological photomicrograph showing a basaloid neoplasm with a cystic component in the dermis (H&E, original magnification 10×). (**B**) Higher magnification of the previous picture showing both basaloid (epithelial) and sarcomatous (mesenchymal) components (H&E, Original Magnification 40×).

A 79-year-old man with a 4-month history of a fast-growing 20 mm nodule on his cheek was seen in a private practice by one of the authors (FR). Histologic sections demonstrated a biphasic tumor with an infiltrative growth pattern with the epithelial component made by nests and trabeculae of basaloid cells with a retraction artifact and central necrosis (Figure 4). On immunohistochemical staining, the cells were positive for CKAE1/AE3. The mesenchymal component was formed by a dense proliferation of spindle cells that were mitotically active and were Vimentin positive. A diagnosis of Basal Cell Carcinosarcoma (BCCS) was made. The patient was lost before a follow-up.

## 3. Discussion

A cCS is a rare and aggressive malignant biphasic tumor, of which approximately 120 cases have been reported in the literature up to the beginning of 2024 [10]. Although rare, the growing number of reported cases has allowed us to add some knowledge to a poorly characterized entity, both from a clinical, histological, and molecular point of view [9,10]. It is important to underscore that there are different names to define it, such as metaplastic carcinoma, sarcomatous carcinoma, pseudosarcoma, and biphasic sarcomatoid carcinoma, reflecting the important and unresolved debate regarding this histogenesis [11,12]. Since cCSs are visible, these lesions may be identified earlier than visceral carcinosarcomas and, consequently, may have a better prognosis [11]. The majority of cases arise on sun-damaged skin, such as the head and neck, and are typically found in older adults with a male predominance [12]. A number of theories have been contributed to explain the development of a cCS. These include the divergence (monoclonal) theory, which holds that the tumor originates from a single progenitor cell that contains populations of mesenchymal and malignant epithelial cells, and the convergence (multiclonal) hypothesis, which holds that the tumor arises from two distinct progenitor cells [11,12]. The latter theory seems to be sustained by a recent paper that used massive parallel genome sequencing [13].

Regarding a pilomatrical CS, the first reported case present in the literature was by Hanly et al. [14] in 1994, in which a 36-year-old male complained of a lesion on his cheek that four months later metastasized to the right upper lobe of the lung. Then, other cases were published [11,15,16,17,18,19,20,21,22], and Leecy et al. reported data about a pilomatrical CS using a comparative genomic hybridization analysis (CGH) on both the carcinomatous and sarcomatous components, showing that each component had many copy number variations (CNVs), with the sarcomatous component having more CNVs than the epithelial elements (twelve vs. five CNVs, respectively). Furthermore, the sarcomatous component also contained a homozygous deletion of chromosome 17q25 and a homozygous loss of chromosome 9p21, which contained the CDKN2A (p16) gene. Certain aberrations were similar, such as the gain on chromosome 20p13–p11.1 and the deletion on chromosome 9p24.1–p13.2, which suggests that the two components shared a common clonal origin [17]. The morphological characteristics of our case are quite similar to those of the cases reported in the literature, also regarding β-catenin immune expression in epithelial and mesenchymal components. Indeed, both the components were positive for cytoplasmic and sometimes nuclear expression, and knowing that β-catenin in a pilomatricoma and pilomatrical carcinoma was able to be induced by mutations in exon 3 of CTNNB1, it could be that these mutations may be contributory to pilomatrical tumors [23]. Regardless, as underscored by both Suemune et al. [22] and Luong et al. [20], the tumor showed no mutation in exons 3, 4, and 5 of the CTNNB1 gene, and Suyama et al. [16] also found no mutation in exon 3 of the CTNNB1 gene. So, to elucidate the significance of aberrant β-catenin expression in pilomatrical carcinosarcomas, additional investigation is necessary [22].

A Merkel cell carcinoma is a rare and aggressive primary cutaneous neuroendocrine neoplasm first described by Toker in 1972 as a trabecular carcinoma of the skin [24]. The incidence is about two–six cases per one million people, and the possibility of divergent differentiation was recognized, with frequent association with an SCC, which can arise as synchronous or metachronous lesions as well as closely intermingled lesions [25,26]. Rarely, the possibility of the presence of sarcomatous component has been described, both homologous and heterologous. Until now, only seven cases of an MCC with sarcomatous differentiation have been published, of which four had rhabdomyoblastic features [27,28,29,30]. Importantly, Adhikari et al. [29] reported a very rare case of components of MCC with rhabdomyoblastic differentiation in a 93-year-old patient with a lesion of the left upper eyelid. In particular, the authors underlined the importance of a correct differential diagnosis with alveolar rhabdomyosarcoma, which may be a morphological mimic of this variant of a MCC. Furthermore, the authors reported for the first time the usefulness of using the antibody against a large T-antigen Polyomavirus of an MCC as a differential diagnostic method compared to other similar entities when molecular biology results were negative for fusion genes typical of alveolar rhabdomyosarcoma. Regarding the most updated hypothesis about the pathogenesis of this variant of an MCC, it seems that Merkel cells arise from an undifferentiated keratinocytic stem cell that would represent the common precursor of both Merkel cells and keratinocytes in the epidermis and adnexal structures, explaining the combination of an MCC with epidermal or adnexal tumors [30]. Furthermore, the multi-differentiative pattern not only has an academic relevance but also poses a diagnostic challenge for the limited cases reported of an MCC with rhabdomyosarcomatous differentiation. Indeed, the presence of a sarcomatous component in an MCC represents a poor prognostic factor, analogous to a sarcomatous component, homologous or heterologous in mixed tumors of other organs [29,30].

A basal cell carcinosarcoma is a rare malignant biphasic tumor comprising distinct basaloid epithelial and mesenchymal components [31], and it is one of the uncommon forms of a cutaneous carcinosarcoma, which also includes a squamous cell carcinosarcoma and cutaneous adnexal carcinosarcoma [32]. Despite only 54 reported cases in the literature, the actual incidence might be higher due to underreporting, discrepancies in tissue processing, and variable histological appearances [33]. A BCCS predominantly occurs in sun-exposed skin areas and mainly affects elderly men [34]. Clinically, these tumors can present as new, rapidly forming masses or as pre-existing lesions that show sudden rapid growth. They vary in size from 0.3 to 15 cm and are often described as exophytic with frequent ulceration, hemorrhage, and crusting, with almost half of the cases occurring on the head, particularly the ear [33,35]. As already mentioned for other kinds of cCSs, several theories exist regarding the origin of a BCCS as follows: (1) Single Stem Cell theory, according to which both sarcomatous and carcinomatous components arise from a single stem cell; (2) Induction theory in which the epithelial component induces the sarcomatous component through close association; (3) Conversion Theory in which a metaplastic transformation of the carcinomatous element gives rise to the sarcomatous component; (4) Pseudosarcomatous Reaction, that should be a reaction to the carcinoma that gives rise to the mesenchymal component; (5) Collision Theory in which two independent neoplasms join together. Today, the conversion theory, supported by various molecular and clinical reports, is the most widely accepted [33].

## 4. Conclusions

A Cutaneous carcinosarcoma (cCS) is a rare and aggressive biphasic tumor with both epithelial and mesenchymal components. Due to its rarity, understanding a cCS is challenging, but our study adds valuable insights by presenting three new cases. The tumor commonly affects older males and arises on sun-exposed skin. Its aggressive nature necessitates prompt diagnosis and treatment to prevent local invasion and metastasis. Further research is needed to clarify its pathogenesis and improve management strategies.

## Data Availability

Data are contained in the manuscript.
